# The closing longevity gap between battery electric vehicles and internal combustion vehicles in Great Britain

**DOI:** 10.1038/s41560-024-01698-1

**Published:** 2025-01-24

**Authors:** Viet Nguyen-Tien, Chengyu Zhang, Eric Strobl, Robert J. R. Elliott

**Affiliations:** 1 Centre for Economic Performance, London School of Economics and Political Science, London, UK; 2 Department of Economics, University of California San Diego, San Diego, CA USA; 3 Department of Economics, University of Birmingham, Birmingham, UK; 4 Department of Economics, University of Bern, Bern, Switzerland

**Keywords:** Economics, Environmental sciences, Engineering, Developing world, Business and management

## Abstract

Electric vehicles are increasingly being adopted in Great Britain and other parts of the world, driven by the perception that they offer a cost-effective alternative to internal combustion engine vehicles while reducing emissions. However, a key element that underpins this perception is the longevity of electric vehicles, which remains relatively under researched. Here we show that although early battery electric vehicles (BEVs) exhibited lower reliability than internal combustion engine vehicles, rapid technological advancements have allowed newer BEVs to achieve comparable lifespans, even under more intensive use. Longevity is also found to be impacted by engine size, location and make of vehicle. We provide parameter estimates for life mileage that can be used to update life cycle assessment and total cost of ownership studies of different vehicle powertrains. Our results also shed light on BEV diffusion patterns, fleet replacement strategies and end-of-life treatment planning, including the increasingly important debate around BEV battery recycling and second-life options.

## Abstract (web summary)

Big data from MOT tests in Great Britain shows that early battery electric vehicles (BEVs) were less reliable than internal combustion vehicles. Here Nguyen-Tien et al. show that rapid technological advancements mean newer BEVs have comparable lifespans, even when more intensively used.

## Main

The electric vehicle (EV) revolution is widely considered as a way to decarbonize the transport sector and to reduce air pollution from tailpipe emissions^1^. To estimate the true environmental benefits of EVs, in particular the fully battery electric vehicles (BEVs), and how they compare with existing petrol and diesel vehicles with internal combustion engines (ICEVs), one needs to consider the entire life cycle of a vehicle and how any benefits are spread across the life cycle.

The production of a typical EV is relatively resource intensive (requiring six times the critical mineral inputs of a conventional vehicle^2^) and has an environmental impact 50% higher than an ICEV^3^. The key argument in favour of an EV transition is that this additional initial environmental cost is more than offset during the use phase if the vehicle has a long enough useful life. Plug-in vehicles such as BEVs offer the opportunity to entirely replace fossil fuels with low-carbon electricity generated from renewable sources such as solar, wind, tidal and geothermal energy. However, if EVs are charged using electricity from coal or gas-fired plants, the environmental benefits could be substantially reduced, resulting in a varied and spatially dispersed impact^4^. Assuming that travel demand remains constant, the current energy mix in Europe means that the longer an EV stays on the road, the greater the environmental benefits^3^. As Europe continues with its own green transition, the energy mix should become increasingly renewable based, making the benefits even larger.

The economic justification for the introduction of policies to promote wider EV adoption is also strengthened if there is a prolonged EV use phase. Putting the environmental impact of production aside, although typically EVs have a higher upfront cost than traditional ICEVs (currently a difference of around US$12,000 according to ref. ^5^), owners tend to benefit from lower operating costs owing to the typically lower cost of electricity compared with gasoline and lower maintenance costs. Argonne National Laboratory^6^ estimate maintenance costs to be US$0.06 per mile for BEVs and US$0.10 per mile for ICEVs. Overall costs may also be reduced further as a result of various policies that improve the financial incentives for purchasing an EV, which range from direct subsidies to reduced or waived road taxes, parking fees and tolls^7–9^. However, policies can also increase costs. For example, the United States recently proposed imposing tariffs of 100% on Chinese-made EVs.

There are several reasons to question the disparity between the expected longevity of EVs and ICEVs. First, emerging technologies, such as EVs, are still in a developmental stage with various new features and configurations, making it uncertain whether they can match the mechanical longevity of ICEVs, which have benefited from decades of continuous research and development and constant marginal improvements. Second, the differences in market structure where the producers of EVs, especially those that are fully electrified, are concentrated in several companies, including new automotive entrants, mean that the new powertrain producers may have different incentives to engage in ‘planned obsolescence’^10^. This refers to a situation where firms with market power produce products with suboptimal quality or durability to maximize profits. Third, early models of new powertrains with high upfront costs may target different subsets of customers^11^ who have different demands for vehicle longevity and exhibit different driving and vehicle care behaviour compared with the average ICE driver. For example, within a household, EVs are often purchased in addition to an existing ICEV^12^.

Having an accurate measure of the longevity of different powertrains, whether the lifetime is measured in time or distance, matters because it is an important input into life cycle assessment (LCA) and total cost of ownership (TCO) models that compare the environmental impact and economic cost between EVs and ICEVs. LCA is a methodology used to assess the environmental impacts of a product or process throughout its entire life cycle and includes raw material extraction, production, use and disposal. See ref. ^13^ for a review and ref. ^14^ for an LCA comparison of EVs and ICEVs. TCO, however, is an estimation of the expenses associated with buying, deploying, using and retiring a product with recent work using this method, including refs. ^15–17^.

Longevity estimates are also important for forecasting automotive sales and planning for end-of-life vehicle treatment. However, knowledge about the longevity of EVs remains relatively limited and what research does exist tends to assume a common functional unit for all EVs and is often extrapolated from an estimate based on the evidence from ICEVs, despite the increasing variety of EVs available on the market, differences in their usage patterns and large technological differences between EVs and ICEVs.

There are two relevant previous studies in this regard. First, ref. ^18^ assumes a common life mileage of 130,000 to compare the emission and cost of 44 hybrid and plug-in hybrid vehicle models in the United States. Second, a recent study commissioned by the UK Department of Transport to assess the environmental impact of a wide range of hybrid and electric vehicles in the United Kingdom assumes that all vehicles stay on the road for 200,000 km (around 124,000 miles) over 14 years^19^. However, the estimates from refs. ^18^ and ^19^ are based on restrictive assumptions owing to the difficulty of accessing data on scrappage rates by powertrain^20^. While there is scrappage data that can provide insights into the longevity of some already scrapped vehicles, this data does not help with estimating future scrappage rates of newer vehicle models, particularly those that use newer technology stacks. The lack of data is most keenly felt for EVs where the main source of information on lifespan is based on lab-based data, expert judgement and educated guesses^21^. It is this gap in knowledge that we attempt to fill in this study.

Our study relates to several strands of literature. Researchers have long been interested in modelling the scrappage and survival rates of petrol and diesel cars^22–24^ as well as the impact of policies aimed at encouraging vehicle scrappage^25–28^. The rise of EVs has also led to a growing interest in understanding the adoption and diffusion of these new powertrains^15,29–32^. So far, however, there has been limited research on estimating the longevity of newer powertrains at the fleet level.

This study makes a unique contribution using the anonymized MOT test dataset, which is increasingly being recognized as a valuable source of big data for addressing different management and socioeconomic issues^20,33^. Here we document that although early BEVs exhibited lower reliability than ICEVs, rapid technological advancements have allowed newer BEVs to achieve comparable lifespans, even under more intensive use. To this end, we demonstrate how big data can be used to better support risk analysis, especially in the transport sector^34^.

## Determinants of vehicle longevity

**Extended Data Fig. 1:**
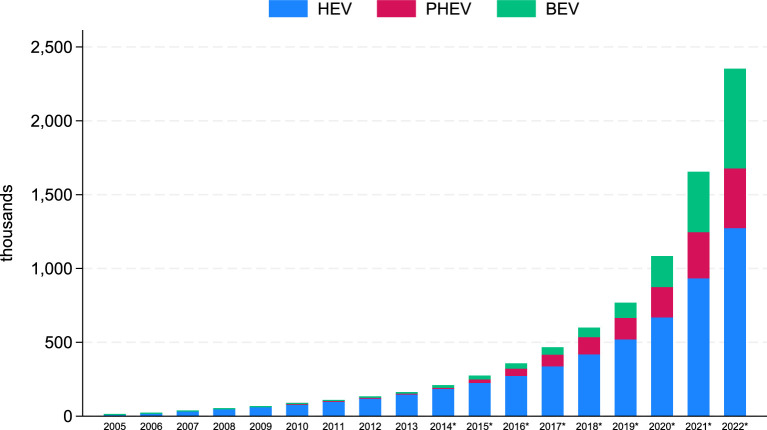
Licensed Electric Vehicles in Great Britain (2005-2013) and United Kingdom (2014-2022). This figure shows the number of licensed electric cars and light goods vehicles by fuel types: hybrid electric vehicles (HEV), plugin electric vehicles (PHEV) and battery electric vehicles (BEV) as recorded in Table 1103 by the Department for Transport (Dft) and Driver and Vehicle Licensing Agency (DVLA). A negligible number of range-extended electric and fuel cell electric are excluded. Data for the United Kingdom is available from 2014. Data from earlier years is for Great Britain. See details in Supplementary Note 1.

The purpose of this paper is to use the compulsory roadworthiness tests (MOT tests) in Great Britain to estimate the longevity of different powertrains with particular emphasis on benchmarking estimates for BEVs against incumbent ICEVs (see Supplementary Note 1 and Extended Data Fig. 1 for the broader context and see Supplementary Note 2 for additional results on hybrid vehicles as presented in a preprint version of this study^35^). To this end, we define vehicle longevity as the service time on British roads, which could end owing to a scrappage decision or export^36^, typically to countries with less stringent environmental regulations and lower operating, maintenance and repair costs. More specifically, statistical analysis on nearly 300 million compulsory MOT tests using STATA and High-Performance Computing allows us to provide timely information on the survival rates of different vehicles, including the newer BEV powertrains, which is information not available from scrappage data alone.

The advantage of using MOT data is twofold. First, as MOT tests are legally required for almost every vehicle on the road, the dataset is comprehensive and represents the actual vehicle fleet. These tests are compulsory for vehicles over 3 years old (or 1 year for certain special vehicles), with exemptions for electric goods vehicles, tractors and historic vehicles, which make up only a small portion of the overall fleet and do not substantially affect the representativeness of our study focused on more recent passenger vehicles. As such, analysing MOT data provides a more holistic view of the fate of the fleet at the end of its life rather than estimates derived from studies that use the small set of vehicles included in commercial survey datasets. Second, anonymized MOT test data are freely available and provide a source of information that is transparent and regularly updated. Unlike other free administrative vehicle registration datasets, anonymized MOT test data includes mileage (odometer reading), cylinder capacity, colour and test location. Mileage data and the date of first registration are particularly important as they allow us to estimate longevity in terms of both years and distance travelled.

The process outlined in the section ‘Data processing’ from over 264 million test results for the period 2005 to 2022 gives us a final population of 29.8 million vehicles as summarized in Supplementary Table 1, which does not include the sample of 371.3 thousand (plug-in) hybrid electric vehicles in Supplementary Note 2. The majority of cars in our sample are petrol (15.1 million) or diesel (14.7 million) and a much smaller number of BEVs (41.6 thousand). This is consistent with official statistics trends in the United Kingdom where petrol and diesel cars still dominate the market despite growing EV sales.

Looking at the cohort variable (the year of first registration) shows that the average petrol car in our sample is slightly older (2010.7) than the average diesel car (2011.3) but also highlights that in contrast the BEVs in our sample are much newer, with an average cohort year of 2015.1. In terms of mileage, unsurprisingly, diesel cars that tend to be used for longer trips have an average mileage of 28.8 miles per day compared with petrol cars, which average 18.2 miles per day. BEVs are somewhere in between these figures averaging around 18.9 miles per day, closer to petrol vehicles. There was a wide range of makes for petrol and diesel vehicles in the dataset, each occupying a small share of the market. However, the choice for electric powertrains is more limited with Nissan (49%), Tesla (19%) and Renault (17%) being the three largest BEV makes.

Supplementary Table 1 also shows that in terms of colour, black followed by silver and then blue are the most popular choices for petrol vehicles while black and then silver and white were more popular among diesel vehicles. Meanwhile white dominated the BEV sample with more than 33% being this colour. In terms of cylinder capacity, a medium engine between 1.0 l and 2.0 l was the most popular across all powertrains (except engine-less BEVs). South East England had the largest population of petrol, diesel and BEVs.

**Fig. 1:**
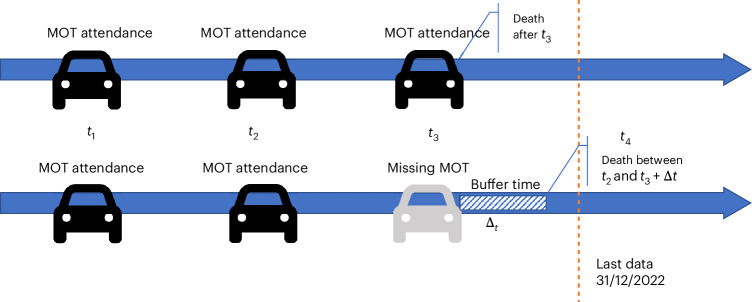
Schema of interval-censored data and the heuristic of death definition. This figure illustrates two types of censored data in our study. Right censored data (upper) consists of vehicles that have attended a recent MOT test, providing information about the ‘survival’ of the vehicle up to that point. Interval censored data (lower) includes vehicles that have missed a recent MOT test, indicating that they may have been retired at some point between the previous MOT test and the expected but missing MOT test. We allow for a ‘buffer period’ after the date the test should have been taken before concluding that a vehicle has been retired to account for delays in taking the MOT.

A concern that one might have using MOT test data to determine a vehicle’s lifespan is that the dataset does not provide the exact date when a vehicle retires. To classify a vehicle as no longer on British roads, extra steps need to be taken. In statistical terms, our dataset includes two types of censored data (Fig. 1). Right censored data consists of vehicles that have attended a recent MOT test, providing information about the ‘survival’ of the vehicle up to that point and interval censored data that includes vehicles that have missed a recent MOT test, indicating that they may have been retired at some point between the previous MOT test and the expected but missing MOT test.

To address these censoring issues, we use a parametric regression model taking into account interval censor mechanism with a Weibull distribution, commonly used to model the survival of vehicles in a fleet. The choice of using a parametric functional form helps greatly enhance computational efficiency, a crucial benefit given the unusually large size of the dataset for this type of nonlinear estimation.

Table 1 presents the results of the survival analysis for three different powertrain categories: petrol (columns 1–3), diesel (columns 4–6) and BEV (columns 7–9). Table 1 shows three specifications for each powertrain using different definitions of retirement for buffers 15, 18 and 21 months (see details in the section ‘The heuristic of death definition’). To enhance interpretability, we present the coefficients in the exponentiated form, capturing hazard ratios. Our preferred specification utilizes the coefficients corresponding to the 18-month cut-off point. Overall, the choice of buffer makes little difference to the sign and significance of the results.

**Table 1:** Survival regressions

	Petrol			Diesel			BEV		
	15m	18m	21m	15m	18m	21m	15m	18m	21m
	(1)	(2)	(3)	(4)	(5)	(6)	(7)	(8)	(9)
Cohort	0.952^***^	0.933^***^	0.930^***^	0.990^***^	0.981^***^	0.982^***^	0.914^***^	0.880^***^	0.879^***^
	(0.0003)	(0.0004)	(0.0004)	(0.0002)	(0.0002)	(0.0002)	(0.01)	(0.01)	(0.01)
Mileage rate (last)	1.082^***^	1.084^***^	1.085^***^	1.063^***^	1.064^***^	1.065^***^	1.021^***^	1.025^***^	1.028^***^
	(0.0003)	(0.0003)	(0.0004)	(0.00006)	(0.00007)	(0.00007)	(0.002)	(0.002)	(0.002)
Under 1.0 l	0.973^***^	0.961^***^	0.959^***^	1.343^***^	1.356^***^	1.370^***^			
	(0.004)	(0.004)	(0.004)	(0.05)	(0.05)	(0.05)			
1.0–2.0 l	1	1	1	1	1	1			
Above 2.0 l	1.068^***^	1.061^***^	1.056^***^	0.798^***^	0.791^***^	0.786^***^			
	(0.004)	(0.004)	(0.004)	(0.001)	(0.001)	(0.001)			
*ρ*	4.069^***^	4.056^***^	4.109^***^	3.406^***^	3.412^***^	3.468^***^	2.507^***^	2.453^***^	2.503^***^
	(0.004)	(0.004)	(0.004)	(0.002)	(0.002)	(0.002)	(0.03)	(0.03)	(0.03)
Region indicators	Yes	Yes	Yes	Yes	Yes	Yes	Yes	Yes	Yes
Colour indicators	Yes	Yes	Yes	Yes	Yes	Yes	Yes	Yes	Yes
Make indicators	Yes	Yes	Yes	Yes	Yes	Yes	Yes	Yes	Yes
Observations	15,131,145	15,131,145	15,131,145	14,685,673	14,685,673	14,685,673	41,640	41,640	41,640
Number of right-censored observations	11,983,876	12,171,912	12,276,601	11,127,288	11,315,899	11,423,605	37,744	38,112	38,265
Number of interval-censored observations	3,147,269	2,959,233	2,854,544	3,558,385	3,369,774	3,262,068	3,896	3,528	3,375
*P* value (*χ*^2^ region)	0.000	0.000	0.000	0.000	0.000	0.000	0.000	0.000	0.000
*P* value (*χ*^2^ colour)	0.000	0.000	0.000	0.000	0.000	0.000	0.006	0.020	0.026
*P* value (*χ*^2^ make)	0.000	0.000	0.000	0.000	0.000	0.000	0.000	0.000	0.000

The exponentiated coefficients and standard errors (in parentheses, not available for reference groups) of baseline survival regressions for petrol (columns 1–3), diesel (columns 4–6) and BEVs (columns 7–9). These regressions include petrol/diesel makes with a minimum of 1,000 vehicles or BEV makes with a minimum of 100 vehicles. Column titles specify the buffer time used to determine the ‘death’ of vehicles, ranging from 15 months, 18 months (preferred), to 21 months. *, ** and *** respectively indicate significance at 0.05, 0.01 and 0.001 levels. The *P* values reported are for two-sided joint Wald tests, which assess whether each set of indicator variables (makes, regions and colours), taken as a whole, are significant.

**Fig. 2:**
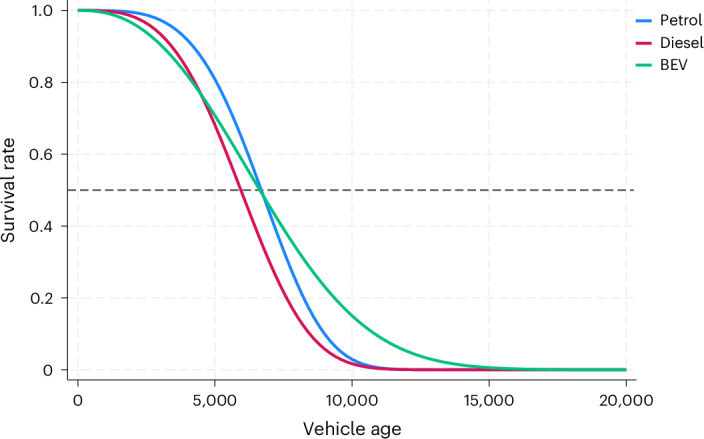
Survival function of different powertrains. The survival function, using the parametric survival estimates from the preferred specifications (18 months) in Table 1, along with the covariate means for three samples: petrol (*N* = 15,131,145), diesel (*N* = 14,685,673) and BEV (*N* = 41,640).

The regressions in Table 1 use a parametric approach and assume a Weibull distribution for the baseline hazard. Estimates of Weibull parameters *ρ* for all powertrains are consistently greater than 1, indicating that the failure rate increases over time. When comparing the 18-month estimates of the *ρ* parameter, the ageing process appears to be more aggressive for petrol vehicles (4.06) and diesel (3.41) than BEVs (2.45). These *ρ* parameters are presented in Fig. 2. The suggestion is that the differences can be attributed to the fact that internal combustion engines have more moving parts and are subject to more wear and tear than electric motors, which are simpler in design.

It is reassuring that for all powertrains, usage patterns appear to be an important predictor of lifespan (significant at the 0.1% level). An increase of 1 mile per day increases the hazard rate by 8.4% for petrol vehicles, 6.4% for diesel and 2.5% for BEVs. This confirms the hypothesis that the more intensively a vehicle is driven, the shorter is its longevity but that BEVs appear to be responding well to increased intensity of use.

Perhaps the most interesting results concern the coefficients for the cohort variable, which are statistically significant and are consistently smaller than 1 for the petrol, diesel and BEV powertrains. A value below 1 implies that over this time period, each of these powertrains has benefited to some extent from technological improvements and that newer models from the same manufacturers exhibit improved reliability over time.

Of these three powertrains, BEVs demonstrate the most rapid improvement, with a 12.0% lower hazard rate for the cohort born 1 year later. By contrast, petrol and diesel vehicles show more modest decreases in hazard rates of 6.7% and 1.9%, respectively. One explanation for these results is that both petrol and diesel powertrains are established technologies that only experience marginal improvements year on year while BEV manufacturers are still on a rapid learning curve. As illustrated in Supplementary Note 4, this finding remains robust when using different sets of covariates and varying thresholds for removing minor BEV makes.

In addition to the previously mentioned variables, MOT data offer a diverse set of details on factors influencing the longevity of vehicles, such as colour, make and location. These categorical variables are incorporated into our model as sets of indicator variables. The Wald *χ*^2^-tests shown in Table 1 confirm that each set of variables collectively significantly correlates with vehicle longevity at the 5% levels and beyond.

**Fig. 3:**
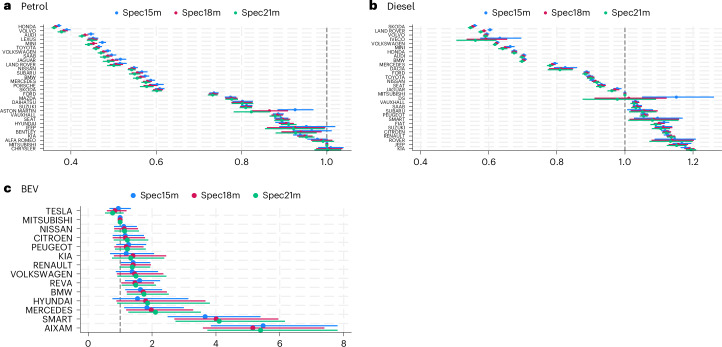
Coefficients and 95% confidence intervals of make indicator variables. **a**–**c**, The exponentiated coefficients and 95% confidence intervals of make indicator variables in Table 1 for three samples (petrol (*N* = 15,131,145 (**a**)), diesel (*N* = 14,685,673 (**b**)) and BEV (*N* = 41,640) (**c**)) and three thresholds (15 months, 18 months (preferred) and 21 months). Coefficients smaller than one (positioned to the left of the dashed vertical lines) indicate brands with lower hazard rates than the reference group (Mitsubishi). For illustrative purposes, **a** and **b** only show the top 30 brands in terms of reliability (with the lowest hazard ratios), while **c** shows all brands included in the regressions.

**Extended Data Fig. 2:**
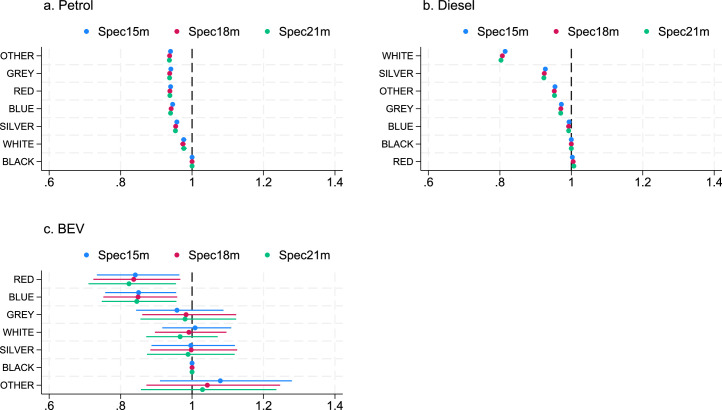
Coefficients and 95% Confidence Intervals of Colour Indicator Variables. This figure illustrates the exponentiated coefficients and 95% confidence intervals of colour indicator variables in Table 1 for three samples: Petrol (panel **a**, N=15,131,145), Diesel (panel **b**, N= 14,685,673), and Battery Electric Vehicle (panel **c**, BEV, N=41,640); and three thresholds 15 months, 18 months (preferred) and 21 months. Coefficients smaller than one (positioned to the left of the vertical lines) indicate colours with lower hazard rates than the reference group (black colour). Less visible confidence intervals for the samples of Petrol and Diesel indicate estimates with near-zero standard errors and high precision. See details in Supplementary Note 3.

**Extended Data Fig. 3:**
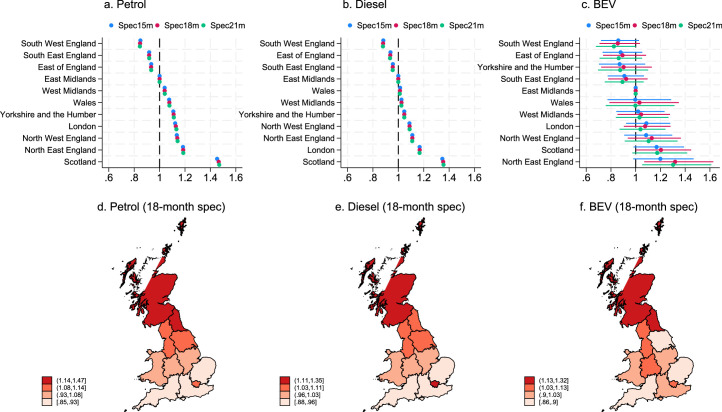
Coefficients and 95% Confidence Intervals of Region Indicator Variables. The upper panels display the exponentiated coefficients and 95% confidence intervals of regional indicator variables in Table 1 for three samples: Petrol (panel **a**, N=15,131,145), Diesel (panel **b**, N= 14,685,673), and Battery Electric Vehicles (BEV) (panel **c**, N=41,640) at three thresholds 15 months, 18 months (preferred) and 21 months. Coefficients smaller than one (positioned to the left of the vertical lines) indicate regions with lower hazard rates than the reference group (East Midlands). Less visible confidence intervals for the samples of Petrol and Diesel indicate estimates with near-zero standard errors and high precision. The lower panels (**d-f**) focus on the exponentiated coefficients from the preferred specification (18-month threshold) for the three samples respectively. See details in Supplementary Note 3. The maps use region boundary data from the Office for National Statistics licensed under the Open Government Licence v.3.0 and contain OS data ⓒ Crown copyright and database right (2024).

Our analysis also reveals a notable variation in vehicle lifespan based on manufacturer. Figure 3 shows the coefficient of hazard ratio for major makes exceeding 100 unique vehicles for BEVs and 1,000 unique cars for other powertrains, relative to the reference make (Mitsubishi). Accordingly, all else equal, relative to the reference group Mitsubishi, the make with the lowest hazard ratio for each powertrain is Honda (petrol), Skoda (diesel) and Tesla (BEV). We only report the top 30 performing makes for petrol and diesel and all makes for BEVs while results for all makes of ICEVs included in our sample are available from the authors upon request. Engine sizes, vehicles’ colour and regions are also significant predictors of hazard rates, as discussed in Supplementary Note 3 and illustrated in Extended Data Figs. 2 and 3.

## Estimated longevity and life mileage

Having considered the other covariates, we now estimate the lifespan of vehicles, which is important for planning fleet replacement and the treatment for the end-of-life of vehicles (for example, organizing scrapping and recycling facilities and hiring skilled labours for these facilities). From a life cycle perspective, the total distance travelled during a vehicle’s lifetime is perhaps more relevant for assessing the emissions of vehicles to provide more information on how driving an EV can offer environmental benefits.

Table 2 presents the estimated median longevity and lifetime mileage for the entire fleet, broken down by powertrain, and the five most popular makes of vehicle. The 18-month specification remains our preferred estimates with the 15- and 21-month results being thought of as providing upper and lower bound estimates.

**Table 2:** Estimated median lifetime and mileage by powertrain and make

	Median lifetime (years)			Median life mileage (miles)			Observations
	15m	18m	21m	15m	18m	21m	
	(1)	(2)	(3)	(4)	(5)	(6)	(7)
	**All vehicles**						
Average	17.1	17.8	17.9	133,076.1	137,567.6	139,021.6	29,858,458
	**Average by powertrain**						
BEV	16.9	18.4	18.6	113,643.4	124,207.8	125,056.2	41,640
Diesel	16.3	16.8	16.9	155,170.3	159,775.1	161,271.6	14,685,673
Petrol	18	18.7	18.9	111,685.8	116,050.7	117,465	15,131,145
	**Average of top five makes by powertrain**						
	**Fully battery electric vehicles**						
TESLA	17.7	20.3	20.9	179,972.9	204,197.9	208,873.4	7,810
HYUNDAI	15.1	15.6	15.4	134,883.8	138,463.5	136,038.1	192
NISSAN	17.4	18.8	18.8	113,252.3	121,771.1	121,263.6	20,449
KIA	17.9	18.5	18.9	113,388.4	116,654.4	118,963.1	438
MERCEDES	15	16.1	15.8	85,405.4	90,926.3	89,193.6	413
	**Diesel**						
SKODA	17.2	17.7	17.9	176,392.3	181,849	183,564.9	316,592
VOLVO	18.1	18.6	18.8	174,806	180,176.9	181,445.2	371,857
LAND ROVER	19.5	20.4	20.6	169,205.7	176,211.5	178,144.9	653,027
VOLKSWAGEN	17.1	17.6	17.8	170,776.8	175,653.2	177,150.6	1,572,090
HONDA	17.7	18.2	18.4	170,581.9	175,141.2	176,501.6	238,388
	**Petrol**						
AUDI	19.9	20.9	21.2	135,997.6	143,100.4	145,168.8	493,401
VOLVO	19.7	20.5	20.7	137,314	142,308.8	144,023.5	70,294
LAND ROVER	18	18.7	18.9	130,251.8	134,874.3	136,626.5	25,756
LEXUS	18	18.4	18.6	130,450.3	133,259.7	134,500.2	33,338
SAAB	17.2	17.6	17.8	130,250.8	132,722.3	134,218	38,591

This table presents the estimated median lifetimes of all vehicles included in the regression sample, using the results shown in Table 1 and a breakdown by powertrain and make. The median life mileages have been estimated from the median lifetime and the mileage rate calculated at the final test of each vehicle then averaged over the sample or subsamples. We prefer the 18-month specification and use the 15-month and 21-month as the lower and upper bounds of our estimates.

When all powertrains are combined, panel A shows that the average vehicle lifetime is 17.8 years and travels about 138,000 miles during this lifetime (columns 2 and 5). This lifetime mileage is close to the 130,000 miles/200,000 km widely used in the LCA literature^18^. A decrease in the buffer time for our assumption of a vehicle’s death leads to a slightly reduced estimate. The 15-month specification suggests an average lifespan of 17.1 years and 133,000 miles travelled, while the 21-month buffer time suggests an average lifespan of 17.9 years and 139,000 miles travelled.

Our lifetime estimates are higher than the average age of a vehicle at scrappage, which was reported as 13.9 years in 2015^37^. There are several reasons for this disparity. First, we provide lifetime estimates for almost every car that has ever joined the fleet, including a large number that are still in operation, rather than conditioning our estimates on those that have already been scrapped. However, we do exclude cars that are scrapped early, for example, owing to accidents within the first few years, preventing them from undergoing their first MOT test at 3 years old. The selection bias means that scrapped cars would have a lower estimated lifetime than surviving cars. Second, our updated analysis focuses on cars registered between 2005 and 2017, with an average registration year of 2011. These are newer models compared with those scrapped in 2015, most of which were likely registered in the early 2000s. Technological advances over the last two decades have contributed to prolong lifespan, as indicated for the majority of vehicles in Table 1. Furthermore, these relatively newer vehicles in our samples are also less susceptible to the major scrappage scheme that was introduced in the 2009 United Kingdom Budget^38^ and concluded in March 2010, which incentivized the scrappage of cars over 10 years old. Finally, reduced vehicle usage, as measured by miles travelled per year, has also contributed to a longer overall lifetime.

Panel B reveals substantial disparities in the lifespan and mileage performance across different powertrains. When comparing petrol and diesel, our baseline estimates indicate that a petrol vehicle survives for 1.9 more years, but covers 44,000 miles less compared with a diesel vehicle. BEVs offer promising characteristics, with an average lifespan of 18.4 years, which approximates that of an average petrol vehicle. Importantly, BEVs surpass petrol cars in terms of lifetime mileage, covering 124,000 miles across their lifetime. Panel C provides a breakdown of lifetime mileage estimates for the leading five brands within each powertrain category. The top-performing BEV make is Tesla, while Skoda and Audi lead the way for diesel and petrol, respectively. While intuitive, these findings contribute to a systematic and granular understanding of reliability in powertrains, including the emerging one.

## Trends in vehicles use and longevity

**Fig. 4:**
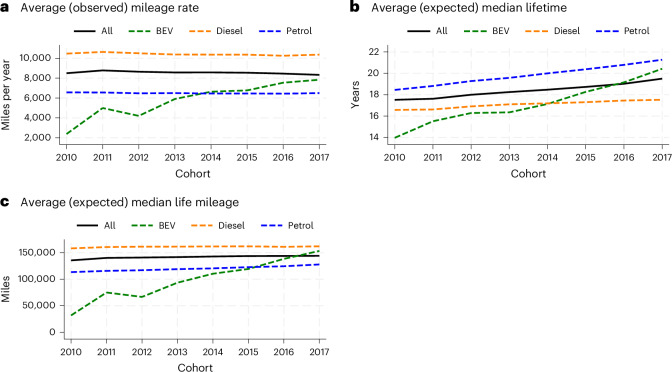
EV revolution. **a**–**c**, Aggregates of the mileage rate (at the last test) (**a**), the median lifetime (**b**) predicted by the 18-month specification illustrated in Table 1, and the lifetime mileage (**c**) as predicted by the two variables mentioned above, categorized by cohort and powertrain.

The next stage is to look at the evolution of the patterns of vehicles use by different powertrains, their expected longevity and miles travelled throughout their life cycle using the predictions generated from our preferred 18-month specifications from Table 1 and odometer information recorded at the last test of each vehicle. The aggregate trend in Fig. 4 captures several factors, including shifts in technology, driver preferences, behaviour and the range of products available in the market.

Figure 4a shows a fairly flat or even declining trend in vehicle usage, measured as miles travelled per year across the entire sample. This decline aligns with a broader reduction in travel demand, as reported in the National Travel Survey, which has a particularly pronounced impact on newer models^39^. BEVs show a substantial increase in usage, with mileage rates increasing from approximately 2,200 miles per year for the 2010 cohort to 7,800 miles per year for the 2017 cohort.

This result can be attributed to the diffusion of BEVs into various segments, including those with higher travel demands, and improvements in technology that have reduced range anxiety. It is important to note that BEV usage patterns may differ from those of ICEVs owing to distinct purchase motivations. Some buyers may choose small-battery, low-range BEVs for inner-city trips or as second vehicles, while others may favour BEVs for higher mileage, as the return on investment improves with greater use. Technological advancements, such as the increase in average BEV range from 79 miles to 151 miles between 2010 and 2017^40^, have made BEVs a viable and attractive choice for individuals who require longer travel distances on a regular basis.

Figure 4b aligns with our survival analysis results presented in the section ‘Determinants of vehicle longevity’, highlighting an increase in the expected median lifespan for all powertrains. Figure 4c reveals analogous trends in the expected median lifetime mileage. Notably, BEVs have experienced rapid improvements and surpassed the average fleet lifetime mileage in 2017.

## Discussion and conclusion

Technological advances, supportive policies and increasing concern for the environment have driven the shift from traditional internal combustion engines towards cleaner powertrains, paving the way towards a net-zero carbon future. To effectively plan for fleet replacement and properly handle retired vehicles in an environmentally friendly manner, a better understanding of vehicle longevity is critical. In light of the shortage of accessible detailed data on vehicle retirement, we propose the use of compulsory MOT test data to track vehicle operation, infer information on its end-of-life and associate it with a wealth of vehicle characteristics recorded during MOT tests.

Our analysis of about 30 million vehicles and nearly 300 million MOT test results uses a Weibull proportional hazard model to identify key predictors of a vehicle’s longevity, including driving intensity, engine size, colour, make and location. The freely accessible data enabled us to conduct a timely evaluation and compare the impact of each determinant among different powertrains, including traditional petrol and diesel engines against newer powertrains such as BEVs.

Our analysis highlights that while BEVs represent a newer technology that was traditionally less reliable, they have rapidly evolved, with the latest BEVs expected to outlast the average ICEVs within the same cohort. This finding necessitates a dynamic approach in assessing the environmental and economic benefits of EVs, rather than assuming a uniform functional unit for all powertrains. Earlier batches of EVs were not only costly but also offered little environmental benefits given their limited lifespan and use. However, if the trends estimated in this study persist, the TCO and environmental advantages of upcoming BEV models could far exceed previous estimations.

However, there are a number of caveats. Government interventions, such as large-scale scrappage schemes largely absent during our study period, could alter patterns of retirement. In addition, it is still early in the ‘S curve’ of EV adoption, and further observation beyond the results presented in this study is needed for newer BEVs, given the variety of technological and business issues that may arise.

Technological developments present both opportunities and challenges. The extended lifespan of EVs may require battery replacements if the original batteries degraded prematurely. Lithium-ion batteries remain the dominant technology for powering EVs and the longevity of these batteries is uncertain^41^. Most new EVs come with warranties of 8 years and 100,000 miles for their batteries^42^ and most research anticipates a lifespan of approximately 8–10 years^43^. Industrial sources tend to be more optimistic about their products, with Tesla claiming that their batteries are designed to outlast the vehicle^44^, and Nissan reporting that almost all of the batteries that they have ever produced are still in use in the EVs they sold over the last 12 years^45^. The analysis of over 6,300 EVs by the fleet management company Geotab suggests that the majority of EV batteries will outlast the usable life of the vehicles they power^46^.

To fully realize the benefits of a longer BEV lifespan, replacement batteries, if necessary, must be affordable relative to the residual value of BEVs without their original batteries. The establishment of a robust circular economy for batteries is imperative to effectively support the dynamics of this technological advancement. As of 2020, the cost to replace a battery ranged from US$4,000 for a 30 kWh Nissan battery to US$10,275 for a 75 kWh Tesla Model 3, compared with US$1,100 to US$3,400 for an ICEV transmission replacement^11^. If battery and replacement costs do not fall quickly enough, owners may choose to prematurely write off their BEVs, which could skew the comparison of BEV longevity against ICE vehicles. This could lead to an overestimation of BEV longevity in our analysis, which TCO and LCA modellers should consider.

In addition, unforeseen technological or business challenges arising later in these vehicles’ lives could disrupt the patterns observed in our model. The widespread adoption of EVs may give rise to new business models such as car leasing and car-hailing. To prevent potential environmental issues, regulation is crucial, as exemplified by the emergence of ‘EV graveyards’ in China^47^, where a substantial number of EVs are left unused before reaching the end of their mechanical lifespan as the new businesses fail. Anecdotal reports on expensive repair costs^48^, high insurance premiums^49^, challenges with battery performance in cold weather^50^ and the increased likelihood of tyre failure^51^ could influence decisions regarding the scrappage of used EVs.

Finally, the quality of MOT data and potential compliance issues, such as incomplete or inaccurate records, delayed testing or failure to get tested, could impact the accuracy of our findings. Although driving with an expired or invalid MOT certificate can result in fines of up to £1,000 and may invalidate an insurance policy, leading to additional penalties, points on the licence or even more severe court action, future research could examine MOT compliance by fuel type and other characteristics, which may help explain some potential biases in the findings of this study. We assume that the robustness of our findings can be strengthened by presenting results under different assumptions and users are advised to use their institutional knowledge to select the results that best suit their analytical purposes. Future research could be enriched by integrating our methodology with data on the export pattern of used EVs from Great Britain^36^, enabling a deeper understanding of scrappage patterns. In addition, the analytical framework in this study could be extended to similar sources of administrative big data available elsewhere or to other sectors, such as heavy-duty goods vehicles, where decarbonization efforts are increasingly important to meet net-zero targets.

## Methods

### Anonymized MOT test dataset

The main dataset used in this study is the anonymized MOT (Ministry of Transport) test database. The MOT test is mandatory for almost all passenger and light-goods vehicles, private buses and motorbikes in the United Kingdom, as required by the Road Traffic Act of 1988. The anonymized MOT test dataset used in this study however only covers tests in Great Britain. To ensure that vehicles are roadworthy and meet minimum environmental requirements, an MOT test must be taken at least once a year for vehicles that are 3 years or older. For certain vehicles, such as taxis, ambulances, and some motor caravans and dual-purpose vehicles, the age at which the first test is required is 1 year. The dataset includes not only information about the time, location and final outcome of the MOT test but also a number of vehicle characteristics. MOT test outcomes were computerized in 2005. As MOT computerization was not fully implemented across Great Britain until 1 April 2006, the dataset is not complete for tests conducted between 1 January 2005 and 31 March 2006. We waited for the May 2023 update, which covers tests from 2005 to 2022, and includes revised 2017 results that were previously missing due to a recording error (corrected in June 2022).

MOT tests are carried out primarily in private garages and by certain local authorities. The locations, known as Vehicle Testing Stations (VTS), are authorized and designated as appropriate by the Driver and Vehicle Standards Agency (DVSA). The VTS and their staff are subject to inspections by the DVSA to ensure that testing is conducted properly using approved equipment. Only specifically approved individuals are permitted to conduct tests, sign official test documents and make database entries. Information about the vehicles, such as the mileage, colour, fuel type and cylinder capacity, is entered or validated by the tester at the time of the test. Vehicles can be tracked using the vehicle ID field, which is based on the registration and vehicle identification number. A high-level postcode region (the first 1–2 digits of the postcode of the VTS) is also provided, but to prevent identifying any individual VTS, any region with fewer than five active sites is merged under the code ‘XX’.

### Data processing

The first stage was to download the MOT test data for each year between 2005 and 2022 from the UK’s Department for Transport (DfT) website and combine them into a single dataset. During the initial cleaning process (Supplementary Table 4), we checked and verified that no records had a missing vehicle ID. As part of data quality control, it was discovered that there were occasional discrepancies in the information provided for the same vehicle in different tests. As a result, rules were established to deal with these inconsistencies. For vehicle types and fuel, information from the most recent test was used, as the classification of vehicles tends to improve over time as testers become more familiar with the new technologies. Information provided in the first test was used for colour and first use time. For cylinder capacity, a majority rule was used and the odometer information and test date from the last test in the dataset was taken to calculate the average mileage of each vehicle throughout its lifetime. Since a car can be brought back for multiple MOT tests on the same day (for example, for retesting), we select the record from the last test day that has the highest non-missing odometer reading. After resolving conflicts in the data, we removed all vehicles that had their first MOT test before it was 2 years old since these vehicles were more likely to be taxis and ambulances. We only analysed Class 4 vehicles that mainly consist of passenger and light-goods vehicles.

The final sample is restricted to four major powertrains: PE (petrol), DI (diesel), EL (electric) or HY (hybrid). We treat electric/hybrid electric (clean) codes (added since 2022) as EL/HY, respectively. While classifying petrol and diesel was straightforward, it was initially necessary to combine EL and HY together as there was no clear and consistent rule to differentiate them, especially in the early years when EVs are much less popular. For example, there were a large number of Toyota Prius (a famous HEV model) and Mitsubishi Outlander (a famous plug-in hybrid electric vehicle (PHEV) model) classified or misclassified as either HY or EL. After an initial pooling, we were then able to split the HY/EL pool into two samples.

First, those with non-missing and non-zero cylinder capacity are put into the (P)HEV sample as they all have an electric motor and an engine (suggested by the cylinder capacity information) and so must be either an HEV or PHEV. Unfortunately, the information provided in the MOT test data did not allow us to differentiate between PHEVs and HEVs so we call this sample (P)HEV. Given this limitation, our primary analysis above focuses on comparing BEVs against petrol and diesel vehicles only. However, Supplementary Note 2 provides some results for this mixed sample of HEVs (which are closer to ICEVs) and PHEVs (which are closer to BEVs).

Second, those with missing or zero cylinder capacity are more likely to have no engine and hence are classified as fully electric vehicles (BEVs). In those cases where vehicles with an engine failed to record an engine size during the MOT test, we consolidated the information on the make and models of these cars and kept only those recognized by the DVSA as BEVs so we did not accidentally include other powertrains. This means that we exclude the small number of (P)HEV vehicles that did not have information on engine size of which the make and model was not recognized by the DVSA as a BEV.

For petrol and diesel cars, we also excluded a negligible fraction of vehicles with missing or zero cylinder capacity. Petrol and diesel were placed into one of the three bins based on cylinder capacity: under 1 l, between 1 l and 2 l, and above 2 l. We dropped the make ‘LONDON TAXIS INT’ and standardized major makes. For example, any vehicles with a make of BMW and other characters (that is, additional details regarding the BMW model) were shortened to just BMW. Similar rules were applied to other makes. We also removed vehicles with unusually high mileages (exceeding 100 miles per day, as recorded at the first/last tests).

Vehicle location was inferred from the postcode area of the first recorded MOT result. Postcodes were then mapped to 11 regions in Great Britain. Relatively aggregated regions were used not only to speed up the computational process but also to allow for easier interpretation since these regions are sufficient to capture some aspects of natural driving patterns, weather conditions and certain socioeconomic characteristics. Vehicles with postcodes coded as ‘XX’ were excluded. Location assumes that owners take the vehicle to a VTS relatively close to where they live.

Finally, a cohort variable was created to capture the vintage of the technology, determined by ‘first use time’ information. Each year is defined as a new cohort and our sample includes vehicles registered in 2005–2017. Cohorts after 2017 are excluded as we want to follow a vehicle for at least two MOT tests from the first test or roughly 5 years from the first use if the vehicle still exists. For sample size reasons, only makes with at least 1,000 unique vehicles for petrol and diesel were included. For BEVs, the threshold was lowered to 100 as this powertrain was still growing from a low base during this period but provides the main motivation for the study. In robustness checks, we also restricted the sample to BEV makes with at least 1,000 vehicles.

### The heuristic of death definition

As the anonymized MOT dataset does not contain explicit information on the retirement of vehicles, we use the date of a vehicle attending an MOT test as evidence of its survival up to that point in time. As our data ends on 31 December 2022, we have a right-censoring issue. More precisely, for a vehicle that regularly attends MOT tests, we do not know the exact date of its death but can conclude that it must have happened after the last MOT test is recorded in the data.

The use of MOT records allows us to infer that death occurred within a certain interval of time. A legal requirement is that if a vehicle is over 3 years old and still operating on British roads, it must attend an MOT test every year. As our database contains all MOT tests taken within our sample period, if a vehicle is not recorded as having taken a test, then it raises questions about the continued survival of that vehicle. If all vehicles strictly follow the legal requirement, we can confidently classify a vehicle as ‘retired’ if no test result is observed for a certain period (usually 1 year) after the last MOT test result recorded in the system.

However, there are a number of practical reasons why a vehicle MOT test may be delayed so we allow for a ‘buffer period’ after the date the test should have been taken before concluding that a vehicle has been retired. For example, some drivers may be unaware of the importance of regular MOT testing or when their MOT is due, particularly if the vehicle recently changed ownership. The cost of an MOT test and any necessary repairs can also be a factor for some owners, particularly if they are facing financial difficulties. Vehicles that are not used frequently or have mechanical issues may be kept off the road until they can be repaired, which can also push back the eventual MOT date that is recorded in the system.

Figure 1 gives an example of an MOT attendance pattern and illustrates the vehicle retirement assumptions used in the analysis. The top line shows that the vehicle regularly attended MOT tests at times *t*_1_, *t*_2_ and *t*_3_. As the cut-off point of our data is the end of 2022, in this case, we do not observe the vehicle fate as the expected MOT *t*_4_ has not yet happened and thus we conclude that the vehicle fails at some point after *t*_3_, or in other words within the interval (*t*_3_, *∞*). However, the second line shows a vehicle that attended regular MOT tests up to *t*_2_ but missed the MOT test that should have happened in *t*_3_. To account for delays in taking the MOT in that year, we allow a buffer Δ*t* and search again. If we do not see the vehicle attending an MOT test within the designated buffer period, we conclude that the vehicle no longer operates on British roads and classify it as retired between the interval (*t*_2_, *t*_3_ + Δ*t*).

The selection of buffer time Δ*t* is an empirical matter. One should note that if we allow for a long Δ*t*, we may miss information on some real deaths of vehicles and lose useful information (that is, classify an interval-censored death as a right-censored death). By contrast, if we assume too short a Δ*t*, we may misclassify some surviving vehicles with late MOT attendance as retired. Our heuristic approach to selecting the appropriate buffer time is to analyse the distribution of the gaps between consecutive MOT test dates in our cleaned database (which includes more than 264 million tests). Our analysis suggests that around 50% of tests, including those impacted by COVID-19 disruptions, fall strictly within a year of the previous MOT test. Recent research indicates that up to 5.2 million cars could be on UK roads without a valid MOT certificate, with 360,000 of these being presented for a new MOT more than a year after their previous certificate had expired^52^. Therefore, setting a buffer time to zero would classify any vehicle that misses an MOT test within 1 year as retired and would be too strong an assumption. By contrast, when we set the baseline buffer time to 6 months, we capture 99% of tests since results show that less than 1% of tests occur more than 6 months after the original due date. As our baseline, we classify as retired any vehicles that fail to attend an MOT test within 18 months of their last recorded test. As a sensitivity check, our results also include estimates based on two alternative thresholds 3 months early and later than our 18-month baseline at 15 and 21 months.

### Survival analysis

To model the longevity of a vehicle, we use survival analysis, a statistical technique that deals with the expected duration of time until an event occurs^53^. More specifically, we are interested in a non-negative random variable *T* representing the lifetime of a vehicle, that is, the duration until retirement (being scrapped or no longer driving on British roads). The distribution of *T* can be characterized by a survival function, *S*(*t*) = *P*(*T* > *t*), which gives the probability that a vehicle will survive past a certain time *t*, and a hazard function, which specifies the probability for a vehicle to be scrapped in the next infinitely small period of time, Δ*t*, conditional on the fact that the vehicle survives to time *t*.

1 $$h(t)={\lim }_{\Delta t\to 0}\frac{P(t < T < t+\Delta t)}{\Delta tS(t)}=\frac{f(t)}{S(t)}=\frac{f(t)}{1-F(t)}$$

In this equation, *f*(*t*) and *F*(*t*) are respectively the density function and the cumulative distribution function and the survival function can be expressed as *S*(*t*) = 1 − *F*(*t*).

Adopting the proportional hazard function, a common approach to model hazard function *h*(*t*), we assume that the hazard function of a vehicle is proportionate to a baseline hazard function, *h*_0*k*_(*t*), and is adjusted by a vector of time-invariant covariates, *x*_*j*_, that is specific to vehicle *j*, and a vector of coefficients, *β*_*k*_. Here we use the subscript *k* to denote the different powertrain types, including petrol, diesel and BEVs, in both the baseline hazard and the vector of coefficients, to clarify that we model the data separately for each type.

2 $${h}_{j}(t)={h}_{0k}(t)\exp ({x}_{j}^{{\prime} }{\beta }_{k})$$

A range of covariates are included in the analysis. (1) We use the mileage rate (MileageRate_*j*_) recorded at the last test date as a proxy for the usage pattern of vehicles hypothesizing that a vehicle driven more often will tend to retire earlier. (2) We include a cohort variable (Cohort_*j*_) as a proxy for the technology available at the time the vehicle is first on the road. (3) For powertrains with internal combustion engines, we include a vector of indicator variables (EngineSize_*j*_) for cylinder capacity to account for the variation in lifespan across engine sizes (1 l and below, 1–2 l, and 2 l and above). (4) We include a vector (Colour_*j*_) to capture the colour of the vehicle as this choice may be correlated with some unobserved traits related to the choice of colour and the characteristics of drivers that may influence driving patterns (refs. ^54,55^ have suggested that the visibility of vehicles may affect their safety). (5) We use the region that the MOT test was taken (Region_*j*_) to proxy regional driving and road conditions. (6) We include a set of vehicle make indicator variables (Make_*j*_) to explain the variation in vehicle popularity, demand for luxury or cost sensitivity and to capture the possibility that the make of a vehicle may also be correlated with driver characteristics. Equation 2 can be expanded as follows, where Greek lowercase characters denote coefficients and Greek uppercase characters denote vectors of coefficients:

3 $$\begin{array}{rcl}{h}_{j}(t)&=&{h}_{0k}(t)\exp \left({\alpha }_{k}+{\gamma }_{k}{\mathrm{MileageRate}}_{j}+{\delta }_{k}{\mathrm{Cohort}}_{j}\right.\\ &&\left.+{\Pi }_{k}{\mathrm{EngineSize}}_{j}+{\Phi }_{k}{\mathrm{Colour}}_{j}+{\Psi }_{k}{\mathrm{Make}}_{j}+{\Omega }_{k}{\mathrm{Region}}_{j}\right)\end{array}$$

Here we do not explicitly model the impact of policies on the scrappage decisions of vehicle owners. Although there was a UK-wide, government-backed scrappage scheme introduced in the 2009 UK Budget^38^, it was terminated in March 2010 and did not target vehicles registered after 2005 (which is the first cohort included in our sample). More recent regional scrappage schemes, including Birmingham (2021), Bristol (2022), London (2023) and Scotland (2023)^56^, had only a negligible effect on the vehicles in our dataset, given their proximity to the end of our study period (2022). As such, the longevity estimates are mainly driven by mechanical ageing, user behaviour, accidents and market factors, rather than explicit policies. Market factors may include various scrappage schemes run by car manufacturers, which typically offer financial incentives to trade in old vehicles for new.

We further assume that the baseline hazard function is parametric and follows a Weibull distribution such that

4 $${h}_{j}(t)={\rho }_{k}{t}^{{\rho }_{k}-1}\exp ({x}_{j}{\beta }_{k})$$

The key implication of this parametric form is that the hazard rate is monotonic and increasing or decreasing over time, depending on whether the shape parameter *ρ*_*k*_ is greater or smaller than 1, respectively. If *ρ*_*k*_ = 1, the hazard rate is constant over time and the Weibull simplifies to an exponential distribution. The parameterization *λ*_*j*_ = exp(*x*_*j*_*β*_*k*_), which is non-negative, time invariant and covariate dependant, scales the baseline hazard rate up or down and is specific to each vehicle^27^. We use the Weibull proportional hazard model as the literature suggests that it is well suited to model the retirement of vehicles with censored data^27,57^. Again, the subscription *k* of *ρ*_*k*_ highlights the fact that our models permit distinct shape parameters across powertrains. Meanwhile, other observable covariates come into play, affecting the scale parameter of the Weibull distributions within each powertrain.

The vector of the coefficient *β* and the shape parameter *ρ* were estimated with maximum likelihood. As discussed above, the observations are either right-censored (*j* ∈ RC) or interval-censored (*j* ∈ IC). This means that we do not observe *t*_*j*_ directly but instead have its lower bound *t*_*lj*_ (the last MOT test the vehicle attended) and the upper bound *t*_*uj*_ for some vehicles that missed a recent MOT test. The log-likelihood function for estimation can be written as follows:

5 $$\log L=\mathop{\sum }\limits_{j\in \mathrm{RC}}\log {S}_{j}({t}_{lj})+\mathop{\sum }\limits_{j\in \mathrm{IC}}\log [{S}_{j}({t}_{lj})-{S}_{j}({t}_{uj})]$$

For each vehicle and standard in the literature, we estimate the median lifetime as the point in time where the survival function reaches a value of 0.5:

6 $${\hat{l}}_{j}=\{t:{\hat{S}}_{j}(t)=0.5\}$$

The median lifetime mileage is then estimated as the product of the estimated median lifespan and the estimated mileage rate $({\hat{r}}_{j})$ recorded in the last MOT test.

7 $${\hat{m}}_{j}={\hat{l}}_{j}\times {\hat{r}}_{j}$$

### Reporting summary

Further information on research design is available in the Nature Portfolio Reporting Summary linked to this article.

## Supplementary information

Supplementary Information Supplementary Notes 1–4 and Tables 1–4.

Reporting Summary

## Data Availability

The raw data (MOT testing data results files) were obtained from the Department for Transport as of 23 May 2023 and are regularly updated at https://www.data.gov.uk/dataset/e3939ef8-30c7-4ca8-9c7c-ad9475cc9b2f/anonymised-mot-tests-and-results. The cleaned datasets are available in the figshare repository (https://figshare.com/s/4eb6d5cdd88922030990?file=49753833)^58^. The repository contains data from the Office for National Statistics licensed under the Open Government Licence v.3.0 and contains OS data ⓒCrown copyright and database right (2024).

## Code availability

Codes are available in the figshare repository (https://figshare.com/s/4eb6d5cdd88922030990?file=49753833)^58^ together with cleaned data.
