# Supplementary material for: The closing longevity gap between battery electric vehicles and internal combustion vehicles in Great Britain
Source: Nat Energy. 2025 Jan 24;10(3):354–64. doi: 10.1038/s41560-024-01698-1 (PMC11936829; doi:10.1038/s41560-024-01698-1)
Supplement: Supplementary file 1 — Supplementary Notes 1–4 and Tables 1–4. [file 41560_2024_1698_MOESM1_ESM.pdf]

nature energy

Supplementary information https://doi.org/10.1038/s41560-024-01698-1

The closing longevity gap between battery electric vehicles and internal combustion vehicles in Great Britain

In the format provided by the authors and unedited

Supplementary Information

Contents

Supplementary Notes 1
1 The Electrification of Vehicles in the UK 1
2 The (Plugin) Hybrid Electric Vehicles Sample 4
3 Other Factors in Estimating Hazard Rates 5
4 Robustness checks 7
Supplementary Tables 8
References 14

Supplementary Notes

1 The Electrification of Vehicles in the UK

As economies transition from agriculture to manufacturing and then to service-based economies, the relationship between economic development and environmental degradation tends to follow an inverse U-shape pattern. More precisely, after reaching a certain turning point, advances in technology and increased environmental awareness slow and then reverse the damage to the environment. This is particularly evident in the UK, where, in recent years, decoupling has seen CO2 emissions decrease by 34.2% despite an increase in GDP of 70.7% (1). Of the remaining emissions, largely as a result of deindustrialisation, the transport sector has become the largest emitter,

accounting for 28% of end user greenhouse gas emissions (2). Therefore, electrification of transport, particularly road transport, is expected to play a crucial role in further decarbonising the economy as vehicles powered by traditional fuels, such as high-emission petrol and diesel, are replaced with cleaner powertrains. However, the magnitude of the environmental benefits associated with electrification depends crucially on the power mix of the grid and more specifically how the contribution of electricity generation from renewables compares to that from burning fossil fuels.

Zero-emission options for the transport sector include BEVs and Fuel cell electric vehicles (FCEVs) (3). Unlike traditional powertrains, both rely solely on an electric motor for propulsion and have no combustion engine. A BEV stores energy for its electric motor in a battery that can be recharged by plugging it into an electrical source, whereas an FCEV uses compressed hydrogen. The main alternative to a BEV is a hybrid vehicle of which there are different types. Hybrid electric vehicles which combine electric batteries and a combustion engine are widely accepted as a transition option as long as full electrification remains economically and technically challenging. Some hybrids have an electric motor that supports the main engine, while other hybrids have an electric motor that can propel the vehicle for a limited range, typically 1-2 miles. A plug-in hybrid can be connected to an external power source to recharge its battery, and both its engine and electric motor can propel the vehicle independently. Finally, Range-Extended Electric Vehicles (REEVs) have an internal combustion engine that does not directly power the vehicle, but recharges the battery that propels the vehicle, much like a BEV.

In this paper, we separate BEVs and ICEVs as well as a hybrid category that

combines HEVs and PHEVs but because of this our main results compare only ICE and BEVs. We were unable to split the hybid category into HEVs and PHEVs due to data availability. We did not analyze FCEVs due to their small sample size, reflecting the lesser importance of these new technologies in the study period. REEVs could be either (P)HEVs or BEVs in our dataset although they are also very small in number and indistinguishable given the available data. REEVs, once considered a practical solution for addressing range anxiety, have become less appealing due to advances in battery technology and an expanded charging network. Results for the hybrid category are discussed in Supplementary Note 2

As can be seen in Extended Data Fig 1, by 2022 the UK stock of electric vehicles, including HEVs, exceeds 2.35 million and accounts for 8% of the entire fleet operating on British roads. Although HEVs still make up the majority of the EV stock, sales of BEVs outsold HEVs for the first time in 2021. From a global perspective, data in 2022 indicate that the UK has the fourth largest PHEV fleet (behind China, the USA, and Germany) and the six largest BEV fleet (behind China, the USA, Germany, France, and Norway) (4). The combined market share of BEVs and PHEVs in the UK's vehicle fleet is 2.8% and ranks twelfth, trailing Norway, Iceland, Sweden, Denmark, the Netherlands, Finland, China, Belgium, Switzerland, Germany, and Austria. The UK law mandates that GHG emissions be reduced to net zero by 2050 leading the government to pledge to end the sale of new petrol and diesel cars and vans by 2030 although this date was pushed back to 2035 in 2023.

2 The (Plugin) Hybrid Electric Vehicles Sample

In an earlier working paper version of this study (5), we included a combined sample of hybrid electric vehicles (HEVs) and plug-in hybrid electric vehicles (PHEVs), denoted as (P)HEV. Again it is important to note that based on make and even model information from the dataset, we were unable to distinguish between plug-in hybrid cars (PHEVs) and traditional hybrid electric vehicles (HEVs). For example, the Toyota Prius, initially a flagship HEV, has seen the PHEV version of this model become more popular over time.

This sample could be heterogenous as these two powertrain types may exhibit significant differences in reliability. An analysis by (6) covering 330,000 vehicles from model years 2000 to 2023 found that PHEVs, which integrate internal combustion engines with electric drives, report 146% more owner-reported problems compared to traditional ICEVs. Conversely, HEVs show a notable 26% reduction in problems compared to conventional counterparts. Therefore, we opted not to include this mixed sample in the main analysis but will discuss some key findings here.

Our baseline estimates indicate that the mixed powertrain (P)HEVs demonstrate notably enhanced longevity and mileage performance, with an average lifespan of 25 years and an expected travel distance exceeding 210,000 miles on average, approximately 50% higher than ICEVs. Japanese and Korean brands including Toyota, Honda and Hyundai leads in terms of both lifespan and life mileages.

However, there's a noticeable decline in (P)HEV longevity over time. This reduction in reliability could be attributed to the growing prevalence of PHEVs in recent

vehicle vintages, which represent a newer and less mature technology. Moreover, as HEVs gain popularity, newer cohorts include more affordable HEV models from the same manufacturers, potentially offsetting some of the loss in reliability. The inclusion of new makes of HEVs with less manufacturing experience could also contribute to the reduced reliability of more recent cohorts.

3 Other Factors in Estimating Hazard Rates

Other factors appear significant predictors of vehicle longevity in Section 'Determinants of Vehicle Longevity'. In terms of engine size, smaller engines are associated with lower hazards for petrol vehicles. Compared to the mid-size engine (1.0-2.0 litres), which is the most popular, a small petrol engine is 3.9% less hazardous, while a large engine above 2.0 litres is 6% more hazardous. The results are reversed for diesel, where a big engine above 2.0 litres is 20.9% less hazardous than the mid-size version. The difference in engine size hazards between petrol and diesel vehicles can be attributed to the way in which the engines operate and the design of the vehicles. Petrol engines tend to be more performance-oriented, thus smaller engines may be designed to be more efficient and reliable to meet the demands of high-performance driving. Conversely, diesel engines are often used in larger vehicles, such as vans and SUVs, and are designed to be more durable and efficient at higher speeds and for longer journeys. Finally, a larger engine may be associated with the luxury end of the product range, although there is not enough information in this dataset to control for the body type of vehicles.

When selecting a colour for their car, consumers may have a variety of reasons.

Colour choice may reflect the personal characteristics, gender, or preferences of the owners, and this choice could depend on culture and context (7). As a matter of fashion, vehicle colour tastes vary over time. Using data at our disposal, it appears that vehicle owners of newer cohorts appear to have switched from colours such as black, blue, and silver to white, grey and red. While there may be little reason to believe in a direct link between colour and mechanical reliability, (8) suggest that light colours may be more visible and thus less subject to passive accidents. However, the relationship between colour and crash tendency could also be confounded by driver traits if safe driving habits are correlated with certain colour preferences (9). After controlling for other variables, our analysis suggests that colour may correlate with lifespan, although the effect varies across powertrains. The results are shown in Extended Data Fig 2.

For petrol, all other colours tend to be more reliable than black, but the colour effect is small in magnitude. This finding is consistent with research using Australian data that suggests black vehicles have the highest crash risk during the daytime (9). White vehicles have a much lower hazard rate than others for the diesel. The BEV sample however shows another trend. Bright and vibrant colours such as red and blue appear statistically more reliable than black with coefficients lower than other monochrome colours (silver, white, and grey).

There are also some survival differences based on location. An analysis of the indicator variable coefficients presented in Extended Data Fig 3 reveals a north-south divide for vehicle reliability. For all three powertrains, Scotland and the northern regions of England (the north-west and north-east) have relatively high hazard ratio coefficients. This observed pattern may be attributed to the comparatively rugged

terrain of these areas, relatively poor road conditions as well as the prevailing cold and wet weather conditions. In addition, the presence of more salt used on the roads that accelerates corrosion and rust, coupled with a higher incidence of potholes (due to worse weather and potentially fewer repairs), means vehicles driven in these regions could explain the lower survival rate. Notably, London stands out negatively for diesel vehicles. The hazard rate in this region is surpassed only by Scotland. The high congestion, low average speeds, and frequent stop-and-go traffic patterns characteristic of London may contribute to the increased wear and tear on diesel powertrains. In addition, the introduction of the Ultra Low Emission Zone (ULEZ) and related regulations in London has imposed strict limitations on the circulation of older diesel-powered vehicles, particularly those not meeting the Euro 6 standard (pre-2014 models). This may have contributed to an increase in the discarding of these vehicles by owners in London.

4 Robustness checks

As a robustness check, Supplementary Table 2 gradually introduces additional covariates until reaching a specification as saturated as Table 1 with the preferred 'death' threshold. The coefficients for cohort variable are quite stable regardless of covariate sets. Across all specifications, all powertrains show technological improvement, with the fastest pace observed in the BEV sample. Hazard rate reduction for the cohort born one year later ranges between 12-13.8% for BEVs, 6.7-8.3% for petrol, and 1.9-2.4% for diesel.

[Supplementary Table 2 about here.]

In another exercise, in Supplementary Table 3, we raise the thresholds for selecting major BEV manufacturers from 100 to 1,000, aligning with the criteria applied to other incumbent fuels. Consequently, only BMW, Nissan, Renault and Tesla BEVs are included in the regressions. The hazard rates of BEVs exhibit a slower reduction over time, yet they continue to outpace other powertrains, declining at a rate of 8% per year.

[Supplementary Table 3 about here.]

Supplementary Tables

Supplementary Table 1: Summary Statistics

	Petrol	Diesel	BEV	All vehicles			
	mean	mean	mean	mean	sd	min	max
VARIABLES	(1)	(2)	(3)	(4)	(5)	(6)	(7)
Mileage rate (last)	18.2	28.8	18.9	23.4	12.1	0.00017	100
Cohort	2010.7	2011.3	2015.1	2011.0	3.82	2005	2017
First colour:							
- BLACK	0.20	0.22	0.18	0.21	0.41	0	1
- BLUE	0.18	0.14	0.12	0.16	0.37	0	1
- GREY	0.12	0.16	0.11	0.14	0.35	0	1
- OTHER	0.053	0.042	0.024	0.047	0.21	0	1
- RED	0.13	0.069	0.12	0.098	0.30	0	1
- SILVER	0.19	0.19	0.11	0.19	0.39	0	1
- WHITE	0.13	0.19	0.33	0.16	0.36	0	1
Cylinder Capacity:							
- 1.0-2.0 l	0.81	0.76	0	0.78	0.41	0	1
- Above 2.0 l	0.052	0.24	0	0.15	0.35	0	1
- Under 1.0 l	0.13	0.00048	0	0.068	0.25	0	1
- Zero/Missing	0	0	1	0.0014	0.037	0	1
Region:							
- East Midlands	0.062	0.070	0.064	0.066	0.25	0	1
- East of England	0.096	0.096	0.091	0.096	0.29	0	1
- London	0.081	0.067	0.16	0.074	0.26	0	1
- North East England	0.047	0.047	0.043	0.047	0.21	0	1
- North West England	0.13	0.12	0.093	0.13	0.33	0	1
- Scotland	0.11	0.11	0.088	0.11	0.31	0	1
- South East England	0.16	0.16	0.20	0.16	0.37	0	1
- South West England	0.082	0.090	0.11	0.086	0.28	0	1
- Wales	0.047	0.049	0.024	0.048	0.21	0	1
- West Midlands	0.092	0.095	0.070	0.093	0.29	0	1
- Yorkshire and the Humber	0.091	0.099	0.058	0.095	0.29	0	1

Supplementary Table 1: Summary Statistics

	Petrol	Diesel	BEV	All vehicles			
	mean	mean	mean	mean	sd	min	max
VARIABLES	(1)	(2)	(3)	(4)	(5)	(6)	(7)
Make:							
- ABARTH	0.0011	0	0	0.00057	0.024	0	1
- AIXAM	0	0	0.0043	0.0000060	0.0025	0	1
- ALFA ROMEO	0.0031	0.0026	0	0.0029	0.053	0	1
- ASTON MARTIN	0.0011	0	0	0.00055	0.023	0	1
- AUDI	0.033	0.066	0	0.049	0.22	0	1
- BENTLEY	0.0011	0	0	0.00055	0.023	0	1
- BMW	0.028	0.077	0.056	0.052	0.22	0	1
- CATERHAM	0.00013	0	0	0.000065	0.0081	0	1
- CF MOTO	0.000072	0	0	0.000037	0.0061	0	1
- CHEVROLET	0.0076	0.0013	0	0.0045	0.067	0	1
- CHRYSLER	0.0013	0.0024	0	0.0018	0.043	0	1
- CI MOTORHOME	0	0.000082	0	0.000040	0.0063	0	1
- CITROEN	0.032	0.046	0.0073	0.039	0.19	0	1
- DACIA	0.0034	0.0036	0	0.0035	0.059	0	1
- DAEWOO	0.00017	0	0	0.000085	0.0092	0	1
- DAIHATSU	0.0014	0	0	0.00069	0.026	0	1
- DODGE	0.00043	0.00053	0	0.00048	0.022	0	1
- DS	0.0011	0.00086	0	0.00099	0.031	0	1
- FERRARI	0.00054	0	0	0.00028	0.017	0	1
- FIAT	0.039	0.015	0	0.027	0.16	0	1
- FORD	0.16	0.14	0	0.15	0.36	0	1
- GREAT WALL	0	0.00013	0	0.000062	0.0078	0	1
- HONDA	0.040	0.016	0	0.028	0.17	0	1
- HYUNDAI	0.032	0.017	0.0046	0.024	0.15	0	1
- INFINITI	0.00010	0.00047	0	0.00029	0.017	0	1
- ISUZU	0	0.0025	0	0.0012	0.035	0	1
- IVECO	0	0.00018	0	0.000087	0.0093	0	1
- JAGUAR	0.0037	0.014	0	0.0088	0.093	0	1
- JEEP	0.00091	0.0029	0	0.0019	0.044	0	1
- KIA	0.024	0.024	0.011	0.024	0.15	0	1
- LAMBORGHINI	0.00014	0	0	0.000071	0.0084	0	1
- LAND ROVER	0.0017	0.044	0	0.023	0.15	0	1
- LDV	0	0.00044	0	0.00022	0.015	0	1
- LEXUS	0.0022	0.0011	0	0.0016	0.041	0	1
- LOTUS	0.00034	0	0	0.00017	0.013	0	1
- MASERATI	0.00036	0.00021	0	0.00029	0.017	0	1
- MAZDA	0.025	0.0093	0	0.017	0.13	0	1
- MCLAREN	0.000088	0	0	0.000045	0.0067	0	1
- MERCEDES	0.022	0.063	0.0099	0.042	0.20	0	1
- MG	0.0018	0.00020	0	0.0010	0.032	0	1
- MICROCAR	0.00012	0	0	0.000060	0.0078	0	1
- MINI	0.031	0.010	0	0.021	0.14	0	1
- MITSUBISHI	0.0055	0.012	0.0045	0.0087	0.093	0	1
- MORGAN	0.00019	0	0	0.000097	0.0099	0	1
- NISSAN	0.053	0.040	0.49	0.047	0.21	0	1
- PERODUA	0.00030	0	0	0.00015	0.012	0	1
- PEUGEOT	0.051	0.053	0.017	0.052	0.22	0	1
- PORSCHE	0.0052	0.0016	0	0.0034	0.058	0	1
- PROTON	0.00071	0	0	0.00036	0.019	0	1
- QUADZILLA	0.000085	0	0	0.000043	0.0065	0	1
- RENAULT	0.042	0.039	0.17	0.041	0.20	0	1
- REVA	0	0	0.018	0.000025	0.0050	0	1

Supplementary Table 1: Summary Statistics

	Petrol	Diesel	BEV	All vehicles			
	mean	mean	mean	mean	sd	min	max
VARIABLES	(1)	(2)	(3)	(4)	(5)	(6)	(7)
- ROLLS ROYCE	0.00016	0	0	0.000080	0.0090	0	1
- ROVER	0.00083	0.00028	0	0.00056	0.024	0	1
- SAAB	0.0026	0.0046	0	0.0035	0.059	0	1
- SEAT	0.017	0.013	0	0.015	0.12	0	1
- SKODA	0.020	0.022	0	0.021	0.14	0	1
- SMART	0.0053	0.00048	0.0097	0.0030	0.054	0	1
- SMC	0.00015	0	0	0.000078	0.0088	0	1
- SSANGYONG	0.00014	0.0012	0	0.00064	0.025	0	1
- SUBARU	0.0029	0.00087	0	0.0019	0.043	0	1
- SUZUKI	0.023	0.0018	0	0.012	0.11	0	1
- TESLA	0	0	0.19	0.00026	0.016	0	1
- TOYOTA	0.049	0.022	0	0.036	0.19	0	1
- VAUXHALL	0.15	0.093	0	0.12	0.33	0	1
- VOLKSWAGEN	0.068	0.11	0.0095	0.087	0.28	0	1
- VOLVO	0.0046	0.025	0	0.015	0.12	0	1
- YAMAHA	0.00012	0	0	0.000060	0.0077	0	1
Observations	15131145	14685673	41640	29858458			

This table provides summary statistics of the key variables associated with different types of powertrains: petrol, diesel and battery electric vehicles (BEVs). We have only included make-by-powertrain data that exceed 1,000 unique vehicles in the original dataset for petrol and diesel, while the threshold is lowered to 100 for BEVs, as they are still new and less popular. To deal with potential discrepancies in the data, we rely on the first test for region, colour and first use time information, and the majority of tests for cylinder capacity and make information. We then use the odometer information and test date from the last test in our dataset to infer the average mileage of each car across its lifetime.

Supplementary Table 2: Robustness check: Technological improvement with fewer covariates

	Petrol			Diesel			BEV		
	(1)	(2)	(3)	(4)	(5)	(6)	(7)	(8)	(9)
Cohort	0.917***	0.930***	0.933***	0.976***	0.978***	0.981***	0.877***	0.862***	0.880***
	(0.0003)	(0.0003)	(0.0004)	(0.0002)	(0.0002)	(0.0002)	(0.005)	(0.007)	(0.01)
Mileage rate (last)		1.081***	1.084***		1.063***	1.064***		1.015***	1.025***
		(0.0003)	(0.0003)		(0.00006)	(0.00007)		(0.002)	(0.002)
Under 1.0 l			0.961***			1.356***			
			(0.004)			(0.05)			
1.0-2.0 l			1			1			
			(.)			(.)			
Above 2.0 l			1.061***			0.791***			
			(0.004)			(0.001)			
ρ	3.571***	3.944***	4.056***	2.903***	3.355***	3.412***	2.394***	2.404***	2.453***
	(0.002)	(0.003)	(0.004)	(0.001)	(0.002)	(0.002)	(0.02)	(0.02)	(0.03)
Region Indicators	No	Yes	Yes	No	Yes	Yes	No	Yes	Yes
Color Indicators	No	No	Yes	No	No	Yes	No	No	Yes
Make Indicators	No	No	Yes	No	No	Yes	No	No	Yes
Observations	15131145	15131145	15131145	14685673	14685673	14685673	41640	41640	41640
# right-censored obs	12171912	12171912	12171912	11315899	11315899	11315899	38112	38112	38112
# interval-censored obs	2959233	2959233	2959233	3369774	3369774	3369774	3528	3528	3528
pvalue (χ^2: Region)		0.000	0.000		0.000	0.000		0.000	0.000
pvalue (χ^2: Colour)			0.000			0.000			0.020
pvalue (χ^2: Make)			0.000			0.000			0.000

This table reports the exponentiated coefficients and standard errors (in parentheses) of baseline survival regressions for Petrol (columns 1-3, Diesel (columns 4-6), and BEVs (columns 7-9). These regressions include petrol/diesel makes with a minimum of 1,000 vehicles or BEV makes with a minimum of 100 vehicles. Column titles specify the buffer time used to determine the "death" of vehicles, ranging from 15 months, 18 months (preferred), to 21 months. *,**, and *** respectively indicate significance at 0.05, 0.01, and 0.001 levels. The p-values reported are for two-sided joint Wald tests, which assess whether each set of indicator variables (makes, regions, colors), taken as a whole, are significant.

Supplementary Table 3: Robustness check: Technological improvement and BEV selection

	BEVs with at least					
	100 vehicles (baseline)			1000 vehicles		
	15m	18m	21m	15m	18m	21m
Cohort	0.914***	0.880***	0.879***	0.958**	0.920***	0.920***
	(0.012)	(0.012)	(0.012)	(0.014)	(0.014)	(0.014)
Mileage rate (last)	1.021***	1.025***	1.028***	1.018***	1.022***	1.025***
	(0.0022)	(0.0023)	(0.0023)	(0.0024)	(0.0026)	(0.0026)
AIXAM	5.803***	6.153***	7.052***			
	(0.82)	(0.91)	(1.07)			
BMW	1.735***	2.057***	2.283***	1.655***	1.964***	2.184***
	(0.17)	(0.21)	(0.25)	(0.16)	(0.21)	(0.24)
CITROEN	1.226	1.397	1.604**			
	(0.20)	(0.24)	(0.28)			
HYUNDAI	1.637	2.149*	2.431**			
	(0.53)	(0.69)	(0.78)			
KIA	1.262	1.673*	1.743*			
	(0.29)	(0.39)	(0.43)			
MERCEDES	1.948***	2.358***	2.754***			
	(0.38)	(0.49)	(0.57)			
MITSUBISHI	1.059	1.193	1.306			
	(0.19)	(0.22)	(0.25)			
NISSAN	1.178*	1.347***	1.490***	1.200**	1.381***	1.531***
	(0.080)	(0.10)	(0.12)	(0.083)	(0.11)	(0.12)
PEUGEOT	1.341*	1.423**	1.606***			
	(0.17)	(0.19)	(0.22)			
RENAULT	1.493***	1.682***	1.804***	1.486***	1.682***	1.810***
	(0.12)	(0.15)	(0.16)	(0.12)	(0.15)	(0.17)
REVA	1.707***	1.746***	1.949***			
	(0.23)	(0.24)	(0.28)			
SMART	3.881***	4.784***	5.359***			
	(0.52)	(0.66)	(0.76)			
TESLA	1	1	1	1	1	1
	(.)	(.)	(.)	(.)	(.)	(.)
VOLKSWAGEN	1.458*	1.736**	1.955***			
	(0.27)	(0.34)	(0.40)			
ρ	2.507***	2.453***	2.503***	2.556***	2.459***	2.511***
	(0.027)	(0.026)	(0.026)	(0.032)	(0.029)	(0.029)
Region Indicators	Yes	Yes	Yes	Yes	Yes	Yes
Color Indicators	Yes	Yes	Yes	Yes	Yes	Yes
Observations	41640	41640	41640	37696	37696	37696
# right-censored obs	37744	38112	38265	34860	35191	35325
# interval-censored obs	3896	3528	3375	2836	2505	2371
pvalue (χ^2: Region)	0.000	0.000	0.000	0.000	0.000	0.000
pvalue (χ^2: Color)	0.006	0.020	0.026	0.001	0.006	0.005

Columns (1) - (3) in this table report the exponentiated coefficients and standard errors (in parentheses) of baseline survival regressions for Battery Electric Vehicles (BEVs), corresponding to columns (7)-(9) in Table 1. These regressions include BEV makes with a minimum of 100 vehicles. Columns (4) - (6) raise the threshold, retaining only major makes with at least 1000 vehicles, similar to incumbent ICEVs in column (1)-(5) of Table 1. Column titles specify the buffer time used to determine the "death" of vehicles, ranging from 15 months, 18 months (preferred), to 21 months. *,**, and *** respectively indicate significance at 0.05, 0.01, and 0.001 levels. The p-values reported are for two-sided joint Wald tests, which assess whether each set of indicator variables (makes, regions, colors), taken as a whole, are significant.

Supplementary Table 4: Cleaning Data

Description	Number of Unique Vehicle ID
Combine tests between 2005-2022	69997828
Select cohort 2005-2017	33934639
Keep class 4 vehicles	31708670
Keep major fuel types	31675292
Drop vehicles with first MOT before 2 years old	31379314
Drop if mile rate exceeds 100 miles/day	30297914
Drop taxis make LONDON TAXIS INT	30289491
Drop postcode XX	30288095
Select major brands by fuel	30245811
Drop non-BEV with missing/zero cylinder capacity	30229738

This table lists the major data cleaning steps. The final sample here includes (P)HEVs mentioned in Supplementary Note 2.

References

[1] Agbugba, G., Okoye, G., Giva, M. & Marlow, J. The decoupling of economic growth from carbon emissions: UK evidence. Tech. Rep. (2019).

[2] Department for Business, Energy & Industrial Strategy. Annex 1: 2020 UK greenhouse gas emissions, final figures by end user and uncertainty estimates (2022). URL https://assets.publishing.service.gov.uk/government/uploads/system/uploads/attachment_data/file/1064962/annex-1-1990-2020-uk-ghg-emissions-final-figures-by-end-user-sector-fuel-uncertainties-estimates.pdf.

[3] HM Government. The Road to Zero Next steps towards cleaner road transport and delivering our Industrial Strategy. Tech. Rep. (2018). URL https://assets.publishing.service.gov.uk/government/uploads/system/uploads/attachment_data/file/739460/road-to-zero.pdf.

[4] IEA. Global EV Data Explorer – Data Tools (2023). URL https://www.iea.org/data-and-statistics/data-tools/global-ev-data-explorer.

[5] Nguyen-Tien, V., Elliott, R. J., Strobl, E. & Zhang, C. Estimating the longevity of electric vehicles: What do 300 million MOT test results tell us? CEP Discussion Papers dp1972, Centre for Economic Performance, LSE (2024). URL https://ideas.repec.org/p/cep/cepdps/dp1972.html.

[6] Consumer Reports. Who Makes the Most Reliable New Cars? (2023). URL https://www.consumerreports.org/cars/car-reliability-owner-satisfaction/who-makes-the-most-reliable-cars-a7824554938/.

[7] Heap, S. H. & Talavera, O. Street-level bureaucracy: best to be grey (or silver) on Friday, in Halifax (2019).

[8] Lardelli-Claret, P. et al. Does Vehicle Color Influence the Risk of Being Passively Involved in a Collision? Epidemiology 13, 721–724 (2002). URL http://journals.lww.com/00001648-200211000-00019.

[9] Newstead, S. & D'Elia, A. Does vehicle colour influence crash risk? Safety Science 48, 1327–1338 (2010). URL https://www.sciencedirect.com/science/article/pii/S0925753510001220.
