# Supplementary material for: The closing longevity gap between battery electric vehicles and internal combustion vehicles in Great Britain
Source: Nat Energy. 2025 Jan 24;10(3):354–64. doi: 10.1038/s41560-024-01698-1 (PMC11936829; doi:10.1038/s41560-024-01698-1)
Supplement: Supplementary file 2 — Reporting Summary [file 41560_2024_1698_MOESM2_ESM.pdf]

# nature portfolio

Corresponding author(s): Viet Nguyen-Tien

Last updated by author(s): 2024/11/16

# Reporting Summary

Nature Portfolio wishes to improve the reproducibility of the work that we publish. This form provides structure for consistency and transparency in reporting. For further information on Nature Portfolio policies, see our Editorial Policies and the Editorial Policy Checklist.

## Statistics

For all statistical analyses, confirm that the following items are present in the figure legend, table legend, main text, or Methods section.

| n/a | Confirmed | |
|---|---|---|
| ☐ | ☒ | The exact sample size ($n$) for each experimental group/condition, given as a discrete number and unit of measurement |
| ☐ | ☒ | A statement on whether measurements were taken from distinct samples or whether the same sample was measured repeatedly |
| ☐ | ☒ | The statistical test(s) used AND whether they are one- or two-sided<br>*Only common tests should be described solely by name; describe more complex techniques in the Methods section.* |
| ☐ | ☒ | A description of all covariates tested |
| ☐ | ☒ | A description of any assumptions or corrections, such as tests of normality and adjustment for multiple comparisons |
| ☐ | ☒ | A full description of the statistical parameters including central tendency (e.g. means) or other basic estimates (e.g. regression coefficient) AND variation (e.g. standard deviation) or associated estimates of uncertainty (e.g. confidence intervals) |
| ☐ | ☒ | For null hypothesis testing, the test statistic (e.g. $F$, $t$, $r$) with confidence intervals, effect sizes, degrees of freedom and $P$ value noted<br>*Give P values as exact values whenever suitable.* |
| ☒ | ☐ | For Bayesian analysis, information on the choice of priors and Markov chain Monte Carlo settings |
| ☒ | ☐ | For hierarchical and complex designs, identification of the appropriate level for tests and full reporting of outcomes |
| ☒ | ☐ | Estimates of effect sizes (e.g. Cohen's $d$, Pearson's $r$), indicating how they were calculated |

*Our web collection on statistics for biologists contains articles on many of the points above.*

## Software and code

Policy information about availability of computer code

| | |
|---|---|
| Data collection | Data from the website (below) was automatically downloaded using STATA version 17.rev2023-01-10 and the High Performance Computing system at the University of Birmingham, UK. |
| Data analysis | Data analysis was made using STATA version 17.rev2023-01-10 with High Performance Computing system at the University of Birmingham, UK and STATA version 18. |

For manuscripts utilizing custom algorithms or software that are central to the research but not yet described in published literature, software must be made available to editors and reviewers. We strongly encourage code deposition in a community repository (e.g. GitHub). See the Nature Portfolio guidelines for submitting code & software for further information.

## Data

Policy information about availability of data

All manuscripts must include a data availability statement. This statement should provide the following information, where applicable:

- Accession codes, unique identifiers, or web links for publicly available datasets
- A description of any restrictions on data availability
- For clinical datasets or third party data, please ensure that the statement adheres to our policy

This research use freely accessible data from UK Department for Transport (https://www.data.gov.uk/dataset/e3939ef8-30c7-4ca8-9c7c-ad9475cc9b2f/anonymised-mot-tests-and-results) and region boundary data from the Office for National Statistics licensed under the Open Government Licence v.3.0.

## Human research participants

Policy information about studies involving human research participants and Sex and Gender in Research.

| | |
|---|---|
| Reporting on sex and gender | N/A |
| Population characteristics | N/A |
| Recruitment | N/A |
| Ethics oversight | N/A |

Note that full information on the approval of the study protocol must also be provided in the manuscript.

# Field-specific reporting

Please select the one below that is the best fit for your research. If you are not sure, read the appropriate sections before making your selection.

☐ Life sciences ☐ Behavioural & social sciences ☒ Ecological, evolutionary & environmental sciences

For a reference copy of the document with all sections, see nature.com/documents/nr-reporting-summary-flat.pdf

# Ecological, evolutionary & environmental sciences study design

All studies must disclose on these points even when the disclosure is negative.

| | |
|---|---|
| Study description | This study estimates the longevity of battery electric vehicle (BEV), petrol and diesel. |
| Research sample | This study uses a dataset of nearly 300 million MOT records from 2005 to 2022 for nearly 30 million vehicles registered in Great Britain. |
| Sampling strategy | We processed the full dataset, applied restriction criteria where needed (see below and the main text). The data sample is large in the nature. |
| Data collection | We downloaded data from UK Department for Transport. |
| Timing and spatial scale | We collected the full set of anonymised MOT tests and results data in 2023. |
| Data exclusions | We excluded hybrid electric vehicles (including plugin versions) as we cannot differentiate them. We've applied some criteria to restrict the main sample (keep recent cohorts and remove outliers in mileages, minor brands for example). There exclusions are listed and carefully justified in the method session. |
| Reproducibility | The research is reproduced using STATA codes. |
| Randomization | No randomization is invovled. |
| Blinding | The original data was anonymised by the provider. |

Did the study involve field work? ☐ Yes ☒ No

# Reporting for specific materials, systems and methods

We require information from authors about some types of materials, experimental systems and methods used in many studies. Here, indicate whether each material, system or method listed is relevant to your study. If you are not sure if a list item applies to your research, read the appropriate section before selecting a response.

## Materials & experimental systems

| n/a | Involved in the study |
|---|---|
| ☒ | ☐ Antibodies |
| ☒ | ☐ Eukaryotic cell lines |
| ☒ | ☐ Palaeontology and archaeology |
| ☒ | ☐ Animals and other organisms |
| ☒ | ☐ Clinical data |
| ☒ | ☐ Dual use research of concern |

## Methods

| n/a | Involved in the study |
|---|---|
| ☒ | ☐ ChIP-seq |
| ☒ | ☐ Flow cytometry |
| ☒ | ☐ MRI-based neuroimaging |
